# 

**Published:** 1879-05

**Authors:** 


### THE AMERICAN JOURNAL OF DENTAL SCIENCE,

EDITED BY

F. J. S. GORGAS, M. D., D. D. S.,

AND

JAMES B. HODGKIN, D. D. S.

VOL. 13.—THIRD SERIES.

SURGEON GEN'L'S OFFICE
LIBRARY.

BALTIMORE:
SNOWDEN & COWMAN.

LONDON:
TRUBNER & CO., 60 PATERNOSTER ROW.

1879—1880.

### INDEX TO VOLUME XIII.---THIRD SERIES.

A National Dental Association, - - - - 359

A New Department, - - - - - - 48

A Guide to Surgical Diagnosis, - - - - 288

A Remarkable Old Man, - - - - - 95

A Very Large Salivary Calculus, - - - - 143

A New Insect Powder, - - - - - - 142

A Perfumed Solution of Iodoform, - - - 144

A Singular Case—Extrusion of a Bicuspid, - - 376

A Question, - - - - - - - 480

A Remarkable Case, - - - - - - 515

A Batch of Dentists, - - - - - 47

Action of Substances on the Teeth, - - - 96

Alveolar Abscess, - - - - - - 455

Abrasion of the Teeth, - - - - - - 468

Amalgams in Children's Teeth, - - - - 189

American Nervousness, Its Philosophy and Treatment, 167, 204

American Academy of Dental Science, - - 274, 472

Almanac, Dental, - - - - - - 48

American Dental Association, - - - - 134

Anæsthesia and Anæsthetics, - - - - - 102

An Anomalous Case of Replantation. - - 191

Animal Grafting, - - - - - - 34

An Odontome, - - - - - - 504

Annual Meetings of Alumni of Baltimore College of

Dental Surgery, - - - - 48, 520

Another Discoverer of Anæsthesia, - - - 526

American Dental Convention, - - - - 135

Applications of Celluloid, - - - - - 123

Artificial Palate, - - - - - - 383

Artificial India Rubber, - - - - - 384

Arsenic in Bismuth, - - - - - - 480

Asay, J. L., M. D., - - - - - - 493

Astringent Mouth Washes, Evils arising from Daily Use of, 30

Atkinson, Wm. H., M. D., D. D. S., - - - - 193

BIBLIOGRAPHICAL NOTICES.

Dr. Adolph Petermann's Dental Almanac for 1879, 48

Transactions of American Dental Association, - 93

Rhymes of Science, - - - - - 93

Eyesight and How to Care for It, - - - 286

Student's Pocket and Medical Lexicon, - • 286

Medical Chemistry, - - - - - - 287

A Guide to Surgical Diagnosis, - - 288

Vegetarianism the Cure for Intemperance, - - 288

The Phosphates in Nutrition, - - - 288

The Origin and Formation of the Dental Fallicle, - 332

The American Agriculturist, - - - 333

Winter and Its Dangers, - - - - 334

St. Maur; An Earl's Wooing, - - - 334

Treatise on Dental Caries, - - - - 379

American Academy of Dental Science, - - 382

The Importance of Preserving the Teeth and Dental Ethnography, - - - - - 426

The Mouth and the Teeth, - - - 427

Physician's Visiting List for 1880, - - - 427

Vick's Floral Guide for 1880, - - - 427

Atlas of Human Anatomy, - - - - 428

The Throat and Voice, - - - - 428

The Scientific American, - - - - 428

Bartholow's Materia Medica, - - - 428

Illustrated Dictionary of Scientific Terms, - - 429

Brain Work and Over-work, - - - 572

Sore Throat; Its Nature, Varieties and Treatment, 573

Petermann's Dental Almanac for 1880, - - 573

Our Homes, - - - - - - 573

A Treatise on Oral Deformities as a Branch of Mechanical Surgery, - - - - 571

Headaches; Their Nature, Cause and Treatment, 572

Baltimore College of Dental Surgery, - - - 520, 523

Bills to Regulate the Practice of Dentistry in Maryland and District of Columbia, - - - - 473

Bacon, Josiah, Death of, - - - - 40

Blake, J. D., M. D., - - - - - 80

Brooklyn Dental Society, - - - - 160

Beard, Geo. M., M. D., - - - - 167, 204

Black, G. V., D. D. S., - - - - 504

Conservative Treatment of the Dental Pulp, - - 1

Chewning, Geo. H., D. D. S., - - - 56

Chloroform in Infantile Convulsions, - - - 80

Chewing Gum, - - - - - - 95

Chloroform Accidents, - - - - - 96

Corundum, - - - - - - - 116

Celluloid, Applications of, - - - - 123

Chromic Pharynzitis, - - - - - 143

Chloroform Narcosis, - - - - - 144

Cysts of the Mouth, - - - - - - 178

Chloral as a Counter-Irritant, - - - - - 240

Copperas and other Disinfectants, - - - - 336

Consumption—Its Treatment, - - - - - 371

Chase, Henry S., M. D., D. D. S., - - - 424

Coyle, John H., D. D. S., - - - - - 455

Cleaning Teeth, - - - - - - 460

Chloral Inebriety, - - - - - - 512

Chloral an Antidote to Strychnine, - - - 527

Chloral, Effects of Long-continued Use of, - - 517

Chloral as an Anæsthetic for Children, - - 574

Death from Chloroform in a Dentist's Chair, - - 574

Dental Caries, - - - - - - 336

Dental Pulp, Conservative Treatment of - - - 1

Dental Pulp, Treatment of, - - - - 49, 98

Dental Associations, 38, 40, 87, 88, 89, 131, 132, 133, 134, 135, 153, 305

Death of Josiah Bacon, - - - - 40

Duplicating Vulcanite Plates, - - - - 174, 524

Dental Physiology, - - - - - 180

Duties of the Dentist, - - - - - - 183

Dental Schools, - - - - - - 234

Diseases of the Nervous System, . - - - - 239

Don't Roll your Manuscripts and other Don'ts, - 285

Demand—Supply, - - - - - - 331

Dental Fallacies, - - - - - - 385

Diseases of the Mouth, - - - - - - 447

Dr. Waters' Statement of the Gardiner Case, - 464

Dentistry in the District of Columbia, - - - - 476

Death from a Tooth in the Trachea, - - 480

Dental Pulp, and Root Filling, - - - 481

Dentiphone and Audiphone, - - - 509

Dies and Taps for Amateurs, - - - - - 426

Dr. J. N. Farrar's Lectures, - - - 425

Dental Department of Vanderbilt University, - - 568

Effects of Tea on the System, - - - - 576

Evils from Daily Use of Astringent Mouth Washes, - 30

Ergotine for Neuralgia, - - - - - 144

Effects of Arsenic upon the Pulp, - - - 202

Edison, T. A., - - - - - - 418

Extrusion of a Bicuspid, - - - - - 376

Effects of Arsenic upon the Body, - - - 479

Extirpation of the Dental Pulp and Root Filling, · 481

Effects of Long-continued Use of Chloral, - - 517

Ether Controversy. Justice—Injustice, - - 398

Farrar, J. N., M. D., - - - - - 30

Fallacies, Dental, - - - - - - - 385

False Diplomas, - - - - - - - 65

Freckles, - - - - - - - 144

Family Taints, - - - - - 224

Filling Teeth—A Few Thoughts on, - - - 274

Fistulæ and Scars of the Cheek, - - - 478

Frequency of Bright's Disease, - - - - 528

From a Preliminary Lecture, - - - 20

Ford, Dr. W. W., - - - - - 183

Fortieth Annual Commencement of Baltimore College of Dental Surgery, - - - - - 520

Grady, Richard, D. D. S., - - - - 1, 289

Goodyear Company's Patent, - - - - 85

Georgia State Dental Society, - - 40, 89, 134, 568

Gardiner Case, - - - - - 328, 464

Glycerine Ointment, - - - - 526

Hodgkin, Prof. J. B., D. D. S., - - 20, 108, 376, 398, 447

Harriman, Geo. B., M. D., D. D. S., - - - 25, 27

Hein, A. M. J., D. D. S., - - - - - 65

How to Care for the Eyes, - - - - - 96

How to Avoid Leaving Scars, - - - - 140

Holmes, Dr. W. R., - - - - - 145, 174

Hand Work, Head Work and Symmetry of Brains, - 236

Heating Metals in Vacuo by the Electric Current, 418

Honors to American Physicians, - - - 477

Hygrometric Properties of Glycerine, - - - 236

Ink on the Carpet, - - - - - 528

Indiana Dental Law, - - - - - 129

Indiana Medical Law, - - - - 136

Iodoform, The Use of, - - - - - 192

Inflammation, - - - - - 193

In the Blood, - - - - - - 284

Ingersoll, L. C., D. D. S., - - - - 481

Japanese on Blood Letting, - - - - 528

Law Regulating the Practice of Dentistry in Georgia, - 569

Lauder, John, D. D. S., - - - - - 414

Lecture of Prof. E. Cutter, M. D., - - - 317

Lectures on Operative Dental Surgery and Therapeutics, 544

Long, Crawford W., Portrait of, - - - 400

Loss and Gain, - - - - - - 137

Mal-Practice Case, - - - - - 94

Maryland and District of Columbia Dental Association, 90, 241, 305, 337, 404, 433

Mattison, Dr. J. B., - - - - - 512

Micrology, No. 1, - - - - - - 25

Micrology, No. 2, - - - - - - 27

Mechanical Dentistry, - - - - - 555

Mouth Washes, - - - - - - 30

Magitot, Dr. E. - - - - - - 34

More Dental Schools, - - - - - 234

Method of Removing Hairs, - - - - - 239

Morphology of the Blood in Syphilis, - - 317

Mills, Geo. A., D. D. S., - - - - 366, 460

Metallurgy and Chemistry of Metals, - - 378

National Dental Association, - - 38, 87, 89, 132

New England Dental Societies, - - - 40, 153

New Sources of Rubber, - - - - - - 188

New Experiments in Anæsthetics, - - - 190

New Thing for Cleansing Instruments, - - - 192

Neuralgia, - - - - - - - 240

Nitrous Oxide in Asthma, - - - - - 335

National Dental Association, - - - - 359

Nickel Plating without a Battery, - - - - 384

New Methods of Mounting Artificial Teeth, - 529

New York Odontological Society, - - - - 519

Obituary Notices.

Brown, Alfred J., Sr., D. D. S., - - - - 92

Traywick, Bryant S., D. D. S., - - - - 92

White, Samuel Stockton, D. D. S., - - - 429

Origin, Progress and Present Condition of Dentistry, - - 56

On General and Local Acidity, - - - - - 68

Orkney Springs, Va., - - - - - 140

On Cysts of the Mouth, - - - - - - 178

Ottofy, Louis, D. D. S., - - - - - 202

Odontological Society of Great Britain, - - 278, 299, 353

Odontome, An, - - - - - - 504

Periostitis, Causes and Remedies, - - - - 145

Premature Decay of Teeth, - - - - - 188

Preliminary Lecture, - - - - - - 20

Poisons and their Antidotes, - - - - 565

Privilege Tax of Dentists, - - - - - - 37

Petermann, Adolph, D. D. S., - - - - 46

Peters, Jno. C., M. D., - - - - - - 68

Personal Similarities and Dissimilarities, - - 141

Poisoning by Chlorate of Potash, - - - - 141

Prevention of Fatal Accidents from Anæsthetics, - 142

Parsons, E., D. D. S., - - - - - - 180

Peters, Samuel, M. D., - - - - - 224

Progress of Dentistry, - - - - - - 289

Patrick, John J. R., D. D. S., - - - - 385

Portrait of Crawford W. Long, - - - - - 400

Preparation of the Mouth for Artificial Dentures, - 414

Pittsburg Dental Association, - - - - - 423

Parramore, T, H., D. D. S., - - - - - 470

Physiology of Saliva, - - - - - - 527

Pathological Changes in the Eyes, - - - 527

Replanted Teeth, - - - - - - - 526

Replanting of Teeth, - - - - - - 528

Rapid Anæsthesia, - - - - - - - 93

Remarks on Foul Breath, - - - - - - 45

Rice, Arthur M., D. D. S., - - - - - - 49

Relations of Dentistry to Medicine, - - - - 108

Recent Experiments with Laughing Gas, - - - 127

Replanting Teeth, - - - - - - - 143

Registration of Dentists in England, - - - - 143

Remedies in Headache, - - - - - - 236

Relations between Age of Mother and Sex of Child, - 383

Reaction of Oral Fluids, - - - - - - 493

Sale of Diplomas, - - - - - - - 539

Strengthening a Weak Tooth by Gold Screws, Etc., - 366

Skin, Inflammation of, - - - - - - - 525

Success of Different Treatment of Typhoid Fever, - 480

Saliva and the Digestion of Starch, - - - - 480

Scott, Martin P., M. D., - - - - - 102, 248

Scientific Hysteria, - - - - - - - 107

Sure Antidote to Poison, - - - - - - 238

Syphilis of the Teeth, Hair, Nails and Skin, - - 248

Sugar and Teeth, - - - - - - - 384

Salisbury, James, M. D., - - - - - 371

Steel Rust, - - - - - - - - 383

Storms and Neuralgia, - - - - - - 139

Teeth Set on Edge, - - - - - - - 238

Tooth-ache as a Cause of Paralysis, - - - - 470

Treatment of Ulcers, - - - - - - 144

Terrible Case, - - - - - - - - 328

Treatment of Consumption, - - - - - 371, 377

Tobacco Smoke, - - - - - - - - 384

Tooth-Caries of Pregnancy, - - - - - - 560

Therapeutical Action of Cold, - - - - - 498

The Audiphone, - - - - - - - 528

Thompson, W H., M. D., - - - - - - 498

The New Departure Again, - - - - - - 424

The Audiphone and Dentiphone, - - - - 509

Thimble Blistering as a Vehicle for Morphia, - 187

The Effects of Tobacco, - - - - - - - 575

Uralium—A New Metal, - - - - - - 525

Ulceration of the Frænum, - - - - - 144

Utah Mineral Wax, - - - - - - - 191

Virginia State Dental Association, - - - - 131

Virchow on the Germ Theory, - - - - - 240

Winder, Prof. R. B., M. D., D. D. S., - - - - 359

Woman Doctor Question, - - - - - - 140

Whooping Cough and Ulceration of Frænum, - - 144

### ARTICLE VIII.

### Dental Associations.

#### *National Dental Association.*

The Eleventh Annual Meeting of this Association, (late "Southern,") will be held in Augusta, Georgia, commencing on the 2nd Tuesday in July, 1879, at 10 A. M.

It is expected that the members of the Georgia, South Carolina and North Carolina State Dental Societies will be present, and a cordial invitation is extended to the profes sion generally.

Officers.

*President.*—Prof. F. J. S. Gorgas, Baltimore, Md.

1*st Vice President.*—Dr. L. D. Carpenter, Atlanta, Ga

*2nd Vice President.*—Dr. J. R. Walker, New Orleans, La
*3rd Vice President.*—Dr. John G. Wayt, Richmond, Va.
*Cor Secretary.*—Dr. A. C Ford, Atlanta, Ga.
*Rec. Secretary.*—Dr. E. S. Chisholm, Tuscaloosa, Ala.
*Treasurer.*—Dr. H. A. Lowrance, Athens, Ga.
*Executive Committee.*—Drs. W. C. Wardlaw, G. H. Winkler, of Augusta; G. W. H. Whitaker, Sandersville, Ga.

STANDING COMMITTEES.

*Physiology and Surgery.*—Drs. Jno. G. Angell, La.; R. B. Winder, Md.; Jas. F. Thompson, Va.

*Dental Education.*—Drs. W. W. H. Thackston, Va.; Jas. F. Knapp, La.; J. Taft, Ohio.

*Histology and Microscopy.*—Drs. S. P. Cutler, Tenn.; W. H. Atkinson, N. Y; Chas. E. Kells, La.

*Dental Chemistry.*—Drs. L. G. Noel, Tenn.; R. Finley Hunt, D. C.; W. T. Arrington, Tenn.

*Dental Therapeutics.*—Dr. S. J. Cobb, Tenn.; G. F. S. Wright, S. C.; E. Floyd, N. C.

*Operative Dentistry.*—Drs. W. C. Wardlaw, Ga.; Jas. H. Harris, Md.; E. L. Hunter, N. C.

*Mechanical Dentistry.*—Drs. Jas. B. Hodgkin, D. C.; Judson B. Wood, Va.; H. M. Grant, Va.

*Dental Literature.*—Drs. J P. H. Brown, Ga.; J. B. Patrick, S. C.; J. Hall Moore, Va.

*Voluntary Essays.*—Drs. S. M. Prothro, Tenn.; Thos. T. Moore, S. C.; G. H. Chewning, Va.; Robt. Arthur, Md.; W. G. Redman, Ky.; E. S. Chisholm, Ala.; W. S. Brown, S. C.; Samuel Rambo, Ala.; Geo. H. Winkler, Ga.; J. G. McAuley, Ala.; W. H. Morgan, Tenn.

The members of the different Standing Committees are earnestly requested to be present, and present papers on the subjects designated. Proper arrangements will be made for reduction of Rail Road fares, &c., by the Executive Committee.

E. S. CHISHOLM,

*Recording Secretary.*

### *Joint Convention of New England Dental Societies.*

A joint Convention of all the Dental Societies in New England, will be held in Wesleyan Hall, Bromfield Street, Boston, June 5th and 6th, 1879.

A large number of essays will be presented by able men of the profession in New England. A cordial invitation is extended to all interested to be present.

ALBION M. DUDLEY,
*Secretary of Committee of Arrangements.*

---

### *Georgia State Dental Society.*

The Eleventh Annual Session of the Georgia State Dental Society, will be held in the city of Augusta, Ga., on Tuesday, the 8th of July next, at 10 o'clock A. M.

The State Board of Dental examiners meets at the same time and place.

L. D. CARPENTER,
*Cor. Secretary.*

*A New Department.*—The readers of the AMERICAN JOURNAL OF DENTAL SCIENCE will be interested and pleased to know that a new department is promised for the future—that of *Micrology* —edited by George B. Harriman, D. D. S., M. D., of Boston. The contributors promise to furnish from month to month, articles, well illustrated, on subjects germain to the profession; and it is confidently expected that the "new department" will be one of the most interesting features of the JOURNAL. Dr. E. Cutter, will work in conjunction with Dr. Harriman, and some new and interesting features of Microscopy may be expected.

H.

---

### BIBLIOGRAPHICAL.

*Dr. Adolph Petermann's Dental Almanac*, 1879. Published by John Alt, in Frankfort on the Main. For sale by all Dental Depots. Price $1.00.

This Almanac contains the names arranged in alphabetical order, and the addresses of more than six hundred dentists practicing in Germany, Austria and Hungary, with two steel engravings of Drs. D. Fricke, Sen., and the late Dr. F. Turnovsky (See Editorial.)

### To Readers and Correspondents.

Communications intended for publication, and exchange journals and papers, should be addressed to the EDITOR OF THE AMERICAN JOURNAL OF DENTAL SCIENCE, 259 N. Eutaw St., Baltimore, Md. All letters relating to the business of the journal, or containing cash remittances, to be directed to Messrs. SNOWDEN & COWMAN. Articles for publication should be received by the editor at least three weeks previous to the first day of the month on which the number in which it is designed for them to appear, is issued.

### CONTENTS:

ARTICLE VIII.—Dental Associations—National Dental Association .......... 38

New England Dental Societies .......... 40

Georgia State Dental Society .......... 40

### EDITORIAL, ETC.

Death of Josiah Bacon .......... 40

Remarks on Article on Foul Breath .......... 45

Dr. Adolph Petermann .......... 46

A Batch of Dentists .......... 47

Annual Meeting of Alumni of Baltimore College of Dental Surgery .......... 48

A New Department .......... 48

### BIBLIOGRAPHICAL.

Dr. Petermann's Dental Almanac for 1879 .......... 48

☞ The American Journal of Dental Science will contain fifty pages of reading matter in each number, making a volume of about

*Six Hundred* pages

**EXCLUSIVE OF ADVERTISEMENTS**

THE

### AMERICAN JOURNAL

OF

### DENTAL SCIENCE.

The *Journal* is issued on the first of each month and contains not less than *fifty* pages of reading matter in each number, exclusive of advertisements, making a volume of *six hundred* pages per annum.

In each number will be found Original Communications, Correspondence, Selected Articles, Monthly Summary, Bibliographical Notices and Editorial Matter, and every effort will be exerted to render the *Journal* worthy of the patronage of the Dental profession.

A generous co-operation is respectfully solicited from the members of the profession; and as the *Journal* has now a printing office of its own, which materially lessens the cost of its publication, it will be issued at the reduced subscription price of

**$2.50 Per Annum in Advance.**

Communications are respectfully solicited on all subjects pertaining to the practice of dentistry, as well as reports of cases occurring in dental practice.

### RATES OF ADVERTISING.

| | 1 MO. | 2 MO. | 3 MO. | 6 MO. | 12 MO. |
|---|---|---|---|---|---|
| 1 PAGE. | $15 00 | $22 50 | $30 00 | $50 00 | $100 00 |
| ½ " | 10 00 | 15 00 | 20 00 | 30 00 | 50 00 |
| ¼ " | 7 00 | 11 00 | 15 00 | 20 00 | 30 00 |
| ⅛ " | 5 00 | 8 00 | 11 00 | 15 00 | 20 00 |

All original communications designed for publication, works for review, new instruments for notice, exchanges, &c., should be directed to the editor, 259 N. Eutaw St., Baltimore, Md.

All advertisements and subscriptions should be addressed to

SNOWDEN & COWMAN,

*Publishers of the Am. Journal of Dental Science.*

86 W. FAYETTE ST. BET. CHARLES & LIBERTY STS.,

BALTIMORE, MD.

